# Noncaseating suppurative granulomatous lymphadenitis in adult onset Still’s disease – a diagnostic dilemma in a tuberculosis-endemic region: a case report

**DOI:** 10.1186/s13256-018-1816-7

**Published:** 2018-10-15

**Authors:** S. W. G. J. W. Chinthaka, R. L. Satarasinghe, S. Senanayake, W. A. P. S. R. Weerarathne, A. A. M. Anfaz, M. P. Deraniyagala

**Affiliations:** Sri Jayewardenepura General Hospital and Post-graduate Training Institute, Sri Jayawardenepura Kotte, Western Province Sri Lanka

**Keywords:** Adult onset Still’s disease, Granulomatous lymphadenitis, Lymphadenopathy

## Abstract

**Background:**

Lymphadenopathy is not an uncommon presentation of adult onset Still’s disease: it is present in up to two thirds of patients with adult onset Still’s disease. The characteristic appearance of lymphadenopathy is described as intense, paracortical immunoblastic hyperplasia. Changes in light microscopy may resemble lymphoma, but immunohistochemistry reveals a benign polyclonal B cell hyperplasia.

**Case presentation:**

We describe a 67-year-old Sri Lankan woman who manifested relapsing prolonged fever, raised inflammatory markers, arthralgia, myalgia, transient skin rash, and cervical lymphadenopathy histologically characterized by noncaseating granulomatous adenitis with central suppuration. Due to the fact of high prevalence of tuberculosis in the region, an extensive diagnostic evaluation was done to exclude the possibility of extrapulmonary tuberculosis; unsuccessful therapeutic trials of complete antituberculosis regime reliably excluded the possibility of tuberculosis and strengthened the diagnostic validity. Disease flares were characterized by systemic inflammatory response syndrome with immediate clinical and laboratory response to corticosteroids. After systematic diagnostic workup which ruled out possible malignant, rheumatic, or autoimmune diseases and infections previously described as causes of granulomatous adenitis, our patient was diagnosed as having adult onset Still’s disease based on Yamaguchi criteria. She required a trial of indomethacin followed by methylprednisolone pulse therapy and long-term maintenance steroid therapy without steroid-sparing immunosuppressive agents or biological disease-modifying antirheumatic drugs. She achieved full disease remission in 3 months. Reevaluation after 6 months and 1 year did not reveal residual disease activity.

**Conclusions:**

To the best of our knowledge this is the first report of suppurative noncaseating granulomatous lymphadenitis attributed to adult onset Still’s disease among Asian or South Asian ethnicities and it is also rarely reported among Europeans and North Americans. It is an extremely challenging situation to diagnose Still’s disease with granulomatous lymphadenitis where tuberculosis is highly prevalent. This case highlights the importance of consideration of adult onset Still’s disease as a potential diagnosis in a compatible clinical context in the presence of noncaseating granulomatous adenitis and indicates that one should not be misled into a diagnosis of tuberculosis by the fact of the high prevalence of tuberculosis, however, the exclusion of other diagnoses is a prerequisite.

## Background

Adult onset Still’s disease (AOSD) is an uncommon inflammatory disorder which has become the eponymous term for systemic onset juvenile idiopathic arthritis [1]. It has a bimodal age distribution at presentation with one peak at ages of 15 to 25 and one peak at ages of 36 to 46 years. There are reported cases in which the age of the patient was more than 70 years [2, 3]. There is no definitive diagnostic test to diagnose Still’s disease rather it is diagnosed by clinical criteria.

Lymphadenopathy was described in more than two thirds of the people with AOSD and characteristically it is described as intense paracortical immunoblastic hyperplasia. Several other histological types have been described but granulomatous lymphadenitis has rarely been reported [2–5]; to the best of our knowledge this is the first reported case in Asia or South Asia.

The differential diagnosis of granulomatous lymphadenopathy is vast; it includes infective diseases (bacterial, viral, fungal, and parasitic), malignant diseases, autoimmune and autoinflammatory disorders, and idiopathic causes like Kikuchi disease and sarcoidosis [5]. Tuberculosis (TB) is a well-known cause of granulomatous suppurative lymphadenitis with caseous necrosis and it is an important differential diagnosis in any patient with granulomatous inflammation; this causes an alternative diagnosis to be extremely difficult in the South Asian region due to the high prevalence of TB [6].

We describe a case of 67-year-old Sri Lankan woman who manifested relapsing prolonged fever, raised inflammatory markers, arthralgia, myalgia, transient skin rash, and cervical lymphadenopathy histologically characterized by noncaseating granulomatous adenitis with central suppuration diagnosed as AOSD after careful exclusion of aforementioned differential diagnoses.

## Case presentation

A 67-year-old Sri Lankan woman was referred by a general practitioner to evaluate high erythrocyte sedimentation rate (ESR) incidentally detected while investigating for acute febrile illness. On admission to the ward she was asymptomatic. There was no history of prolonged fever, altered bowel habits, myalgia, or arthralgia; there was no history of backache, or urinary or bowel symptoms. No significant weight changes or change in appetite were noted. A symptomatic evaluation and systemic review was found to be normal.

She had been previously diagnosed as having hypertension, dyslipidemia, bronchial asthma, and osteoarthritis of bilateral knee joints. Her prescribed medication was rosuvastatin 5 mg taken at night with hydrochlorothiazide 25 mg taken in the morning, and glucosamine sulfate preparation and Ecosprin (aspirin) 100 mg taken at night. Two years before this presentation she presented to a surgical department with a history of painful neck lump and was found to have cervical lymph adenopathy which was biopsied under local anesthesia. The histological appearance favored granulomatous inflammation without caseation. Atypical mycobacterial infection/fungal granulomata/TB with superadded pyogenic infection were considered for the differential diagnoses. She was given category 1 antiTB medications and managed as TB lymphadenitis; treatment continued for 6 months and was completed in liaison with a pulmonologist.

During the current admission a complete blood count (CBC) showed evidence of mild anemia. Her hemoglobin level was 9.1 g/dl; her mean corpuscular volume (MCV) was 86.5 fl, mean corpuscular hemoglobin (MCH) was 27.8 pg, and mean corpuscular hemoglobin concentration (MCHC) was 31.4 g/dl. A direct blood film examination showed normocytic normochromic, mildly hypochromic microcytic anemia. Mild anisopoikilocytosis was noted. Her white blood cell count (WBC) was normal in number and found to have lymphocytes predominance. However, there were no features of lymphoproliferative disorder. Mild eosinophilia and plasmacytoid lymphocytes were seen. Platelet count and morphology was normal. Her ESR was 102 mm/hour and was persistently high throughout the period of evaluation. Her C-reactive protein (CRP) was within normal range. Baseline liver and renal profiles were normal. Urine Bence Jones proteins were negative. A skeletal survey did not reveal any abnormality. A chest X-ray was normal and sputum for acid-fast bacilli (AFB) was negative. Lactate dehydrogenase level in serum was normal. Serum protein electrophoresis indicated elevated alpha fraction, beta 2 fraction, and polyclonal increase of gamma fraction. Urine analysis had evidence of pyuria but culture was sterile. Urine for AFB was negative. As she was asymptomatic and screening was negative she was discharged from the ward and followed up as an out-patient.

A month after discharge she presented with fever and recurrence of lymphadenopathy to a local chest clinic and a biopsy revealed noncaseating granulomata. Immunohistochemistry did not reveal any evidence of hematological malignancy. A TB polymerase chain reaction (PCR) was negative. She was started on category 1 anti-TB treatment.

While on antiTB treatment for 2 months she was admitted to this hospital with history of symmetrical arthritis of bilateral hands and legs, persisting intermittent low grade fever without chills or rigors, and backache. She complained that both large and small joints were painful and swollen. She was diaphoretic and had vomiting, severe loss of appetite, and excessive sweating. She did not have any history of rashes. She had no history of jaundice.

On examination she was febrile, diaphoretic. Bilateral extremity edema was noted but her joints were not inflamed. Abdominal, chest, cardiac, and neurological examinations were normal.

Her ESR was 130; in CBC, her WBC was 12,000 with 82% neutrophils. Her CRP was 200. Her creatine phosphokinase (CPK) level was normal. Possible sepsis was suspected and she was started on intravenously administered ceftriaxone. Her serum calcium level, acetylcholine esterase level, and a magnetic resonance imaging (MRI) of her whole spine were normal. Baseline renal and liver profiles were normal except for mild elevation of transaminases. AOSD, sarcoidosis, polymyalgia rheumatic spectrum disorder, and remitting seronegative symmetrical synovitis with pitting edema (RS3PE) syndrome were taken in a differential diagnosis and further evaluated. A cerebrospinal fluid (CSF) full report did not show any evidence of neurosarcoidosis, TB PCR/culture was negative, and purified protein derivative (PPD) skin test was less than 5 mm. Her antinuclear antibody (ANA)/rheumatoid factor (RF) was negative. Extractable nuclear antigen profile including anti-U1 ribonucleoprotein (RNP) was negative. CSF, serial blood cultures, and serial urine cultures were sterile. Peripheral blood rapid infection detection and PCR amplification for known bacterial and fungal species were done and found to be negative for trace DNA/RNA. Serum ferritin was mildly elevated. Antibiotics were changed in liaison with microbiology team into meropenem and vancomycin and continued for 10 days without success. Persistent fever with raised inflammatory markers was noted even after 3 weeks. Transthoracic and transesophageal echocardiography were normal. Hepatitis serology, retroviral screening, antibody against cytomegalovirus (CMV), and Epstein–Barr virus (EBV) were done and found to be negative. Contrast-enhanced computed tomography (CECT) of her chest and abdomen was done and no evidence of TB or fungal infections was identified. Upper gastrointestinal endoscopy, colonoscopy, and serial ultrasonography were normal.

After extensive diagnostic workup, finally we came to a conclusion and she was diagnosed based on Yamaguchi criteria as having AOSD presenting with granulomatous lymphadenopathy; she was started on intravenously administered methylprednisolone pulse therapy 500 mg, 250 mg, and 250 mg in 3 consecutive days followed by low-dose maintenance steroids and her condition remarkably improved. Her disease remission was characterized by resolving lymphadenopathy, normalization of inflammatory markers, and dramatically improving clinical symptoms. She was discharged for follow-up as an out-patient as she was in disease remission. She was reevaluated after 6 months and 1 year and reported no additional problems and she was doing her normal everyday life.

## Discussion

Here we describe a woman of 67 years which is an unusual age of presentation of AOSD; AOSD is usually expected in two peaks of ages from 15 to 25 and 36 to 46 years [2, 3]. She presented with prolonged high grade fever, relapsing granulomatous lymphadenopathy without caseation, arthralgia lasting more than 2 weeks, abnormal liver function studies manifested with elevation of transaminases and bilirubin, negative ANA and RF, which fulfilled five required features in Yamaguchi criteria including two major criteria. Diagnostic validity was increased by negative infection screening, absence of evidence of malignancies especially lymphoma, and absence of evidence of other rheumatological disorder that would mimic AOSD [7, 8].

In Table 1 we highlight the comprehensive list of differential diagnoses for granulomatous lymphadenitis [5–11]. In Table 2 we apply the Yamaguchi clinical criteria to our patient.

**Table 1:** Causes of granulomatous and/or necrotizing lymphadenitis, with or without suppuration

Viruses	Bacteria	Fungal
EBV CMV HBV HCV HSV adenovirus HIV	*Yersinia*, *Bartonella* *Tropheryma whipplei* *Francisella tularensis* *Brucella* Spirochaetes *Treponema pallidum* *Chlamydia* *Lymphogranuloma venereum* *Rickettsia* *Coxiella burnetii* *Mycobacteria* *Mycobacterium tuberculosis* Atypical mycobacterial infection *Mycobacterium leprae*	Histoplasmosis Aspergillosis Coccidioidomycosis Cryptococcosis
Parasite	Neoplastic	Autoimmune
Toxoplasmosis Leishmaniasis	Hodgkin disease Non-Hodgkin disease, metastatic carcinoma Langerhans cell histiocytosis Seminoma Dysgerminoma	Systemic lupus erythematosus Granulomatosis with polyangiitis Churg–Strauss syndrome Celiac disease, Crohn’s disease Primary biliary cirrhosis, Kawasaki disease
Autoinflammatory and idiopathic		
Necrotic sarcoid granulomatosis Kikuchi-Fujimoto disease Familial Mediterranean fever Hyperimmunoglobulinemia D syndrome PFAPA syndrome		

*CMV* cytomegalovirus, *EBV* Epstein–Barr virus, *HBV* hepatitis B virus, *HCV* hepatitis C virus, *HSV* herpes simplex virus, *PFAPA* periodic fever, aphthous stomatitis, pharyngitis, and cervical adenitis

**Table 2:** Application of Yamaguchi clinical criteria in presented patient

Yamaguchi criteria	Patient’s characteristics
Major Yamaguchi criteria	
Fever of at least 39 °C lasting at least 1 week	+
Arthralgias or arthritis lasting 2 weeks or longer	+
Typical rash (maculopapular, nonpruritic) during febrile episodes	–
Leukocytosis (10,000/μL or greater) with at least 80% granulocytes	–
Minor Yamaguchi criteria	
Sore throat	–
Lymphadenopathy	+
Hepatomegaly or splenomegaly	–
Abnormal liver function studies	+
Negative antinuclear antibodies and rheumatoid factor	+
Exclusions	
Infection	Done
Malignancies: mainly lymphoproliferative	Done
Other rheumatological disorders	Done

Yamaguchi criteria require the presence of five features, with at least two being major diagnostic criteria

Each of the conditions has a different histological appearance and none have pathognomonic description. In the presence of granulomatous lymphadenitis, a clinician should look for wide possibilities of differential diagnoses ranging from infective, inflammatory, and malignancy to miscellaneous causes. Lymph node biopsy is not a prerequisite to diagnose AOSD; however, it helped in differentiating the other possible etiologies in our patient as she presented with asymptomatic lymphadenopathy at initial consultation.

In our patient serial sterile blood, urine, and CSF cultures, as well as negative peripheral blood PCR amplification of bacterial or fungal deoxyriboneucleic acid (DNA), and negative serology of known viral etiologies, reliably excluded the possibility of infective causes. However, because of atypical presentation of known infectious etiologies and extremely diverse presentation of TB in the region, a therapeutic trial of antiTB medication was given and an absence of clinical response excluded the possibility of TB in our patient. The emerging multidrug-resistant TB (MDR-TB) in the region is a possible cause for the therapeutic failure of antiTB regime but the clinical course of the illness and therapeutic response to steroids are incompatible with MDR-TB [12].

Negative extractable ANA screening, negative RF, and absence of clinical criteria to diagnose alternative rheumatological diseases strengthened the diagnosis of AOSD.

Lymphoproliferative malignancies were excluded by the histological appearance of the lymph node biopsy and immunohistochemical studies. However, not performing a bone marrow biopsy due to absence of consent from the patient would have affected the reliability of the exclusion of the above if there was no rapid therapeutic response and no maintaining of the remission of disease with steroid alone.

Kikuchi disease was excluded based on prolonged and relapsing nature of the disease; normal gastrointestinal endoscopies in the absence of other supportive clinical evidence excluded the possibility of Crohn’s disease [13]. Kawasaki disease, which has been described as a cause of granulomatous lymphadenopathy, was incompatible with the clinical course as well as the age of presentation.

However, sarcoidosis is an important differential diagnosis in this presentation. Although classically associated with elevated angiotensin-converting enzyme (ACE) level, hypercalcemia, and non-necrotizing granulomatous lymphadenopathy, it has been previously described as having wide variations in its clinical presentation [9].

Familial Mediterranean fever also described as possible etiology of granulomatous lymphadenitis but due to epidemiological reasons it was not evaluated in our patient which is a possible limitation in the case study [10].

The presentation of our patient was atypical. Lymphadenopathy was the first presentation and over the course of the illness she had leukocytosis with neutrophil predominance, hyperferritinemia, hepatosplenomegaly as well as the typical rash was absent. The aforementioned extensive diagnostic workup reliably excluded the other differential diagnoses and the therapeutic response and compatible clinical criteria validated the diagnosis. Yamaguchi criteria were described to diagnose AOSD with over 93% sensitivity [5].

## Conclusions

To the best of our knowledge this is the first reported case of suppurative noncaseating granulomatous lymphadenitis attributed to AOSD among Asian or South Asian ethnicities and it is also rarely reported among Europeans and North Americans. It is an extremely challenging situation to diagnose Still’s disease with granulomatous lymphadenitis where TB is highly prevalent. The case highlights the importance of considering AOSD in the differential diagnosis of compatible clinical context and not to be misled by the fact of high TB prevalence and emerging MDR-TB in the region in the presence of therapeutic failure.

## Authors’ information

All authors affiliated to Sri Jayewardenepura General Hospital and Post-graduate Institute, Sri Lanka at the time of presentation and evaluation of this patient.
